# Cyclometalation of lanthanum(iii) based MOF for catalytic hydrogenation of carbon dioxide to formate†

**DOI:** 10.1039/c9ra09938g

**Published:** 2020-01-22

**Authors:** Piwai Tshuma, Banothile C. E. Makhubela, Lars Öhrström, Susan A. Bourne, Nabanita Chatterjee, Isaac N. Beas, James Darkwa, Gift Mehlana

**Affiliations:** Department of Chemical Technology, Faculty of Science and Technology, Midlands State University Private Bag 9055, Senga Road Gweru Zimbabwe; Department of Chemistry, Faculty of Science, University of Johannesburg, Kingsway Campus: C2 Lab 328 Auckland Park 2006 South Africa; Chalmers University of Technology, Department of Chemistry and Chemical Engineering, Physical Chemistry Room 9029 Göteborg Sweden; University of Cape Town, Department of Chemistry, Faculty of Science PD Hahn Building 7701 Rondebosch Cape Town South Africa; Department of Natural Resources and Materials, Botswana Institute of Technology Research and Innovation Maranyane House Private Bag 0082 Gaborone Botswana mehlanag@staff.msu.ac.zw

## Abstract

The hydrogenation of carbon dioxide (CO_2_) to formic acid is of great importance due to its useful properties in the chemical industry. In this work, we have prepared a novel metal–organic framework (MOF), JMS-1, using bipyridyl dicarboxylate linkers, with molecular formula [La_2_(bpdc)_3_(DMF)_3_]_*n*_. Network analysis of JMS-1 revealed a new 7-connected topology (**zaz**). The MOF backbone of the activated phase (JMS-1a) was functionalized by cyclometalation using [RuCl_2_(*p*-cymene)]_2_ to produce Ru(ii)@JMS-1a. Both JMS-1a and Ru(ii)@JMS-1a were able to convert CO_2_ in the presence of hydrogen to formate. Ru(ii)@JMS-1a displayed outstanding conversion evidenced by a yield of 98% of formate under optimized conditions of total pressure 50 bar (CO_2_/H_2_ = 1 : 4, temperature 110 °C, time 24 h, 5 mmol KOH, 8 mL ethanol). This work is significant in providing new strategies of incorporating active catalytic centres in MOFs for efficient and selective conversion of CO_2_ to formate.

## Abstract 2

A novel metal–organic framework JMS-1 with rare topology **zaz** shows catalytic activity towards conversion of carbon dioxide to formate.

## Graphical abstract

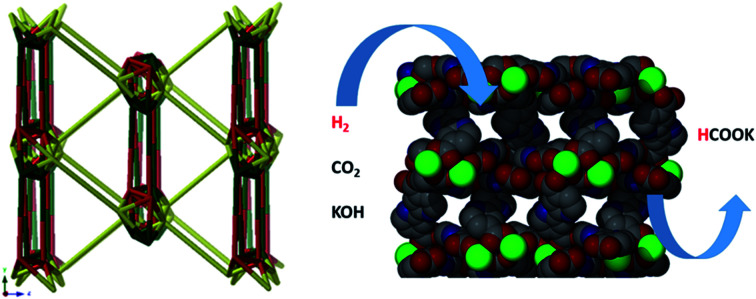

## Introduction

The conversion of carbon dioxide to high value chemicals is of global interest owing to the dangers posed by this greenhouse gas to the environment. Carbon dioxide is also considered as a cheap and renewable source of carbon and its conversion to formic acid is of interest to the chemical industry. The world production of formic acid is around 700 000 tons per year, mostly from combining methanol and carbon monoxide.^1^ In addition to current use, one can view formic acid produced from renewable H_2_ and CO_2_ as a way to store energy.^2,3^

During the past decades extensive research on conversion of carbon dioxide to formate or methanol has been accomplished using both homogeneous catalysts and heterogeneous catalysts.^4–6^ The challenge with using homogenous catalysts, which prove to operate under more milder conditions than their heterogeneous counterparts, is the separation of formic acid or formate adducts from the catalyst and the reaction media. This is because the homogeneous catalyst present in the reaction media can easily convert the produced formic acid/formate adduct back to CO_2_ and H_2_ during the product isolation process. In light of this, it is of paramount importance to design and develop heterogeneous catalysts which can be easily secluded *via* simple filtration prior to the product separation step and can be continuously recycled for successive runs.

Metal–organic frameworks (MOFs) are a new class of crystalline materials which have generated much interest, owing to their potential applications in gas storage,^7^ separation,^8^ catalysis,^9^ sensing^10^*etc.* The often porous and highly ordered nature of these structures provides a unique platform to extend their applications as multifunctional materials. These materials can in principle be tailored to achieve desirable properties and it has been shown that MOFs are capable of capturing and storing gases such as CO_2_ (ref. 11 and 12) and H_2_.^13^ Several strategies have been developed to enhance the volumetric uptake of these gases by MOFs such as the introduction of open metal sites as well as including the amine groups within the MOF pores to enhance the uptake of CO_2_.^14^

Recently, there has been a growing interest in the use of MOFs for conversion of CO_2_ to useful chemicals. The majority of these studies have been focusing on water stable Zr-based MOFs,^12,15–21^ with the catalytically active centres introduced to the MOF backbone by post synthetic metalation of a bipyridine linker. Interestingly, none of these publications report on the activity of the parent MOF towards reduction of CO_2_ but rather focus on the metals introduced into the pores of the MOF such as ruthenium, rhodium and iridium.

In this contribution, we have prepared a La(iii)-based MOF, JMS-1 (Johannesburg and Midlands State) constructed from bipyridyl decarboxylate linkers. Topological analysis of the network revealed a hitherto unobserved 3D net. The MOF's structural integrity changed slightly upon encapsulation of [Ru(ii)Cl(*p*-cymene)] complex through activation of the C–H bond which indicates framework flexibility.^22^ Catalytic studies showed that JMS-1a and Ru(ii)@JMS-1 converted CO_2_ to formate. Our results demonstrate that incorporation of the [Ru(ii)Cl(*p*-cymene)] complex into the MOF enhances the yield of formate produced.

## Experimental section

### Materials and methods

All materials used in this study were of analytical grade and used without further purification. Lanthanum chloride (LaCl_3_·7H_2_O), was purchased from Sigma-Aldrich and used as received.

### Preparation of [La_2_(bpdc)_3_(DMF)_4_]_*n*_ (JMS-1)

2,2′-Bipyridine-4,4′-dicarboxylic acid (H_2_bpdc) (29 mg, 0.113 mmol) and LaCl_3_·7H_2_O (110 mg, 0.327 mmol) were mixed in 11 mL of DMF. The reaction mixture was stirred for 15 minutes and heated in an oven at 110 °C for 48 h. Colorless dendrite-like crystals were obtained.

### Single crystal structure determination

#### Single Crystal data collection

Data collection was performed by single crystal X-ray diffraction using a Bruker KAPPA APEX II DUO diffractometer with graphite monochoromated Mo–Kα radiation (*λ* = 0.71073 Å). The data collections were carried out at low temperature (173 K) using a Cryostream cooler (Oxford Cryosystems UK). Unit cell refinement and data reduction were performed using the program SAINT.^23^ Data were corrected for Lorentz-polarization effects and for absorption (program SADABS). Structure solutions were achieved by direct methods (program SHELXS)^24^ and refined by full-matrix least-squares on *F*^2^ with anisotropic thermal parameters for all non-hydrogen atoms using SHELXL^24^ within the X-SEED^25*a*^ interface. The non-hydrogen atoms were located in the difference electron density maps and were refined anisotropically, while all the hydrogen atoms were placed with geometric constraints and refined with isotropic temperature factors. Due to high solvent disorder some atoms of the DMF molecules were refined isotropically. Restrains on bond distances and thermal motion of disordered groups have been applied (DFIX). The crystal data and refinement parameters are detailed in Table 1. The structure was deposited at the Cambridge Crystallographic Data Centre and allocated the numbers: CCDC 1851044.

**Table 1:** Crystal Data and refinement parameters

JMS-1	
Empirical formula	C_45_H_39_La_2_N_9_O_15_
Formula weight	1223.67
Temperature/K	173(2)
Crystal system	Orthorhombic
Space group	*Pna*2_1_
*a*/Å	18.0826(12)
*b*/Å	13.3216(9)
*c*/Å	21.9623(15)
Volume/Å^3^	5290.5(6)
*Z*	4
*ρ*_calc_ g/cm^−3^	1.5361
*F*(000)	2388
Crystal size (mm)	0.111 × 0.05 × 0.07
Index ranges (*h*, *k*, *l* max)	23, 17, 28
Reflections collected	47 417
Two theta range	1.788–27.556
Independent reflections	11 053
Goodness-of-fit on *F*^2^	1.036
Final *R* indexes [*I* ≧ 2*σ*(*I*)]	0.0546
Final *R* indexes [all data]	0.1596
Largest diff. peak/hole/eÅ^−3^	1.08/−1.00

#### Powder X-ray diffraction (PXRD)

Powder diffraction patterns were measured on a Bruker D8 Advance X-ray diffractometer operating in a DaVinci geometry equipped with a Lynxeye detector using a CuKα-radiation (*λ* = 1.5406 Å). X-rays were generated by an accelerating voltage of 30 kV and a current of 40 mA. A receiving slit of 0.6 mm and a primary and secondary slit of 2.5 mm were used. Samples were placed on a zero-background sample holder and scanned over a range of 4° to 40° in 2*θ* with a step size of 0.01° per second. The XSEED program was used to obtain calculated PXRD patterns from the corresponding single crystal data.

#### Thermogravimetric analysis (TGA)

Thermogravimetric experiments were carried out using a TA Discovery Instrument TA-Q550. In a typical run 1–5 mg of the sample was dried on a filter paper, placed in open aluminium pans and heated in a dry air atmosphere of nitrogen (50 mL min^−1^) at a heating rate of 10 °C min^−1^ within a temperature range of 25–500 °C.

#### FT-IR

Spectra (4000–400 cm^−1^) were obtained neat using a Thermo Scientific Nicolet 6700 FTIR spectrometer equipped with an Attenuated Total Reflectance accessory (ATR) with a diamond crystal.

### NMR studies

^1^H NMR spectra were recorded on a Bruker Ultrashield 400 MHz spectrometer. Spectrometer values were reported relative to the internal standard tetramethylsilane (*δ* 0 : 00). All chemical shifts were reported in ppm.

### Topological analysis

The network topologies discussed in this article were obtained using the free software programs SYSTRE,^25*b*^ and TOPOS PRO,^25*c*^ and we discuss the topologies using the three-letter symbols in the web-based and free Reticular Chemistry Structural Resource database, RCSR.^25*d*^

### Preparation of JMS-1a and Ru(ii)@JMS-1a

JMS-1 was activated by soaking the as-made crystals in methanol for 24 h to allow for the exchange of DMF with a low boiling solvent. The soaked crystals were then heated under a vacuum for 24 h at 78 °C to give the activated phase JMS-1a. Complete removal of the solvent molecules was confirmed by TGA analysis in Fig. S1† which showed no weight loss until decomposition. Cyclometalation of the activated JMS-1a was carried according to a method reported in literature for a homogeneous system.^26^ It is a direct method to produce stable cyclometallated ruthenium(ii) complexes with [RuCl_2_(*p*-cymene)]_2_ precursor, *via* the C–H bond deprotonation in the presence of sodium acetate at room temperature in methanol. In a typical experiment, 50 mg of the activated crystalline powder was soaked in 10 mL methanolic and sodium acetate solution of [RuCl_2_(*p*-cymene)]_2_ for 24 h at room temperature. Successful preparation of Ru(ii)@JMS-1a was confirmed by XPS studies. The amount of Ru present in Ru(ii)@JMS-1a was determined by ICP-OES and found to be 0.4% by weight. Elemental analysis of JMS-1a found % C 43.15, % N 8.45, % H 1.75 calculated, % C 43.05, % N 8.37, % H 1.79: Ru(ii)@JMS-1a, found % C 44.89, % N 7.88, % H 1.98. The increase in the percentage carbon content in Ru(ii)@JMS-1a suggests the presence of *p*-cymene in the functionalized MOF.

### Gas sorption measurements

Porosity and surface properties of the samples were analyzed using Micromeritics ASAP 2460 surface and porosity analyzer. Prior to analysis, the samples were degassed using nitrogen gases (N_2_) at 90 °C for 12 hours, then at 150 °C for 8 h. Thereafter, the samples were weighed and analyzed at 77 K using N_2_ as a probe gas.

### Catalytic studies

In a typical experiment the catalysts were charged into a Teflon-lined stainless-steel reactor equipped 5 mmol KOH in 8 mL ethanol. The reactor was first purged with nitrogen gas three times to remove air from the vessel. This was followed by pressurizing the reactor with CO_2_ and hydrogen (CO_2_/H_2_ = 1 : 1 to 1 : 3) at room temperature. The catalytic reactions were carried out within a temperature range of 90 °C to 120 °C while stirring was maintained at 945 rpm. Upon completion of the reaction, the reactor was cooled to room temperature, the pressure was released before the product was collected into the sample vial. The yield of formate produced was calculated as moles of formate produced divided by the moles of the base used (see example 1 in the ESI†).

The solid catalyst was washed several times with ethanol and dried in an oven at 100 °C for recycling. The reproducibility of the catalytic results was confirmed by repeated experiments.

## Results and discussion

### Structural description

**Fig. 1:**
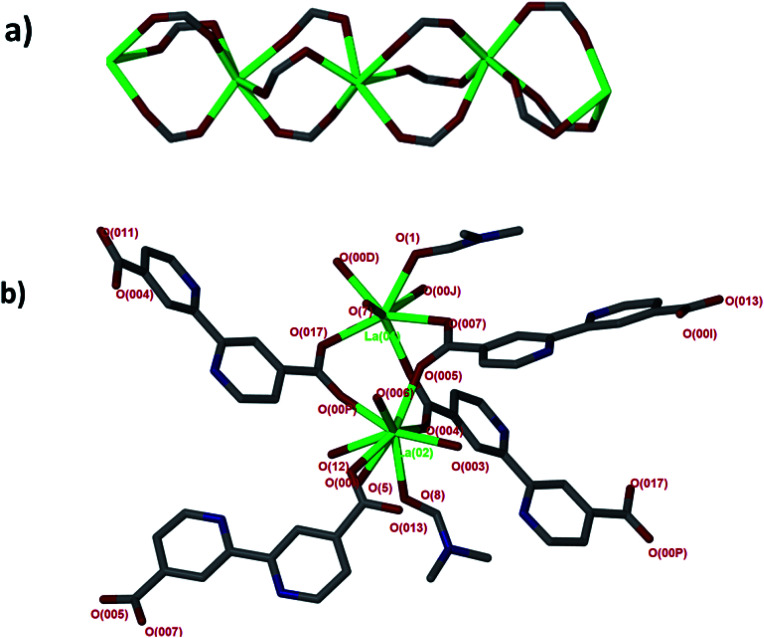
(a) SBU rod generated *in situ*, (b) coordination environment around the two La(iii) modelled in the asymmetric unit.

Single crystal X-ray diffraction revealed that JMS-1 crystallises in the orthorhombic crystal system and space group *Pna*2_1_. In the asymmetric unit of JMS-1, we modelled three bpdc linkers, two crystallographic independent La(iii) centres and a total of three DMF molecules. The structure of JMS-1 is made up of La_2_C_3_O_6_ secondary building unit (SBU) rod which grow along the *b*-axis (Fig. 1a). The SBUs are connected by the bpdc linkers which propagates along the *a*-axis to give a three-dimensional structure. The void space found in JMS-1 is reduced by the presence of the bpdc linkers which connects the SBU along the *b*–*c* plane. Each La(iii) is coordinated to two DMF molecules and six oxygen atoms of the bpdc linker to furnish a square antiprismatic geometry as illustrated in Fig. 1b. One of the coordinated DMF molecules on each of La(1) and La(2) was found to be disordered over two positions and the atoms were refined isotopically. The second coordinated DMF on each of the metal ions in the asymmetric unit was modelled with 50% site occupancy as suggested by TGA analysis. Due to high temperature factors, the atoms of these DMF molecules were also refined isotopically. The bpdc linker assumes a bridging bidentate mode connecting two La(iii) metal ions. The La–O bond distances ranges from 2.41(13) to 2.660(4) Å.

**Fig. 2:**
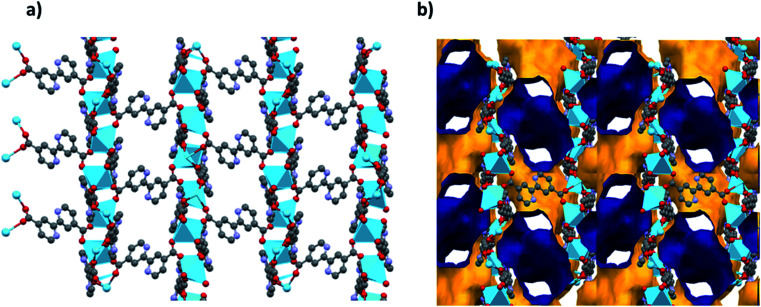
(a) Packing diagram of JMS-1 viewed along the *b*-axis drawn in (a) and stick form and (b) the voids found in JMS-1, the outer and inner surface is shown in yellow and blue respectively.

Analysis of the channels (window dimensions of approximately 6.8 × 5.2 Å) found in JMS-1 which run along the *b*-axis by PLATON^27^ shows that they occupy 41% of the unit cell volume. These channels are decorated by DMF molecules that are coordinated to the La(iii) metal ion. Fig. 2 illustrates the channels found in JMS-1.

**Fig. 3:**
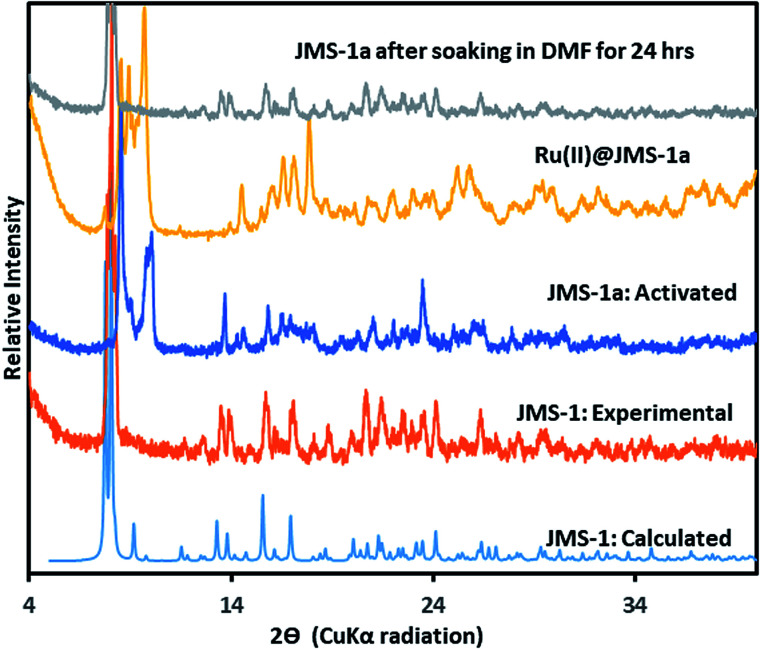
PXRD patterns of JMS-1: calculated, JMS-1: experimental, JMS-1a: activated and the [Ru(ii)*p*-cymene] loaded MOF (Ru(ii)@JMS-1a).

Thermal analysis of JMS-1by TGA (Fig. S1†) shows a 16.5% weight loss between 150 and 250 °C which corresponds to loss of three DMF molecules modelled in the crystal structure of JMS-1 (calculated 17.89). The TGA thermogram is featureless between 320 and 450 °C an indication of high thermal stability. Decomposition of the framework is observed above 460 °C. The phase purity of JMS-1 was confirmed from the excellent agreement between the experimental PXRD pattern of JMS-1 and the calculated PXRD pattern from the single crystal structure (Fig. 3).

The fact that compound JMS-1a has coordinated DMF molecules which can be removed by guest exchange followed by thermal treatment makes it a suitable candidate for catalysis. Removal of coordinated guest molecules has been reported to provide free open metal sites which enhances adsorption of hydrogen and carbon dioxide in the pores.^28,29^ JMS-1a and Ru(ii)@JMS-1a are thermally stable (Fig. S1†) which makes them good catalysts within the temperature range at which catalysis is carried out.

### Topological analysis of the network found in JMS-1

MOFs based on rods, that is when there is a polynuclear coordination entity propagating infinity in one dimension and connecting to other rods by bridging ligands, (infinite metal SBU:s) has received attention lately.^30,31^ One reason is that their topologies do not generally allow for interpenetration of a second network, and another is that they often display “breathing” properties (for example of the MIL-53 types), *i.e.* they may change volume on uptake or discharge of guest molecules.^32^ Compared to the topologies of MOFs based on finite metal SBU:s or single metal nodes that often display one of the high symmetry nets,^33,34^ rod based MOF:s have an intriguing diversity. One recent example is a bismuth based MOF that, although not built of anything more complex than 1,3,5-benzenetricarboxylate (BTC3) ligands and Bi^3+^ ions, has a unprecedented topological complexity with 54 unique nodes and 135 edges, that is the net has transitivity 54 135.^35^

**Fig. 4:**
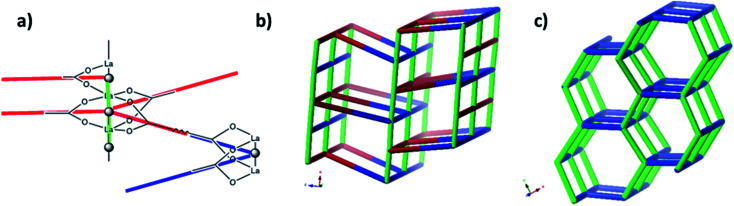
(a) Node assignment for a simple featureless rod with bridging ligands. (b) The resulting **soe**-net in the structure. (c) The most symmetric form of the **soe**-net, the edges corresponding to the rod emphasized in green. The **soe**-net has transitivity 1 4 (1 unique node and 4 unique edges). This description disregards the topology of the rod and the relations of the links this enforces and is not the preferred description of the structure.

A simple approach considers the La-carboxylate rod in compound JMS-1 as a featureless rod, except for the links to the bridging ligands. We then consider rod-centered nodes at the centroids of the tris-carboxylate binding sites. This gives a five-connected **soe**-net as seen in Fig. 4.

This conspicuously looks like the same approach that we use with for example paddle-wheel four-connected SBU:s like the [Cu_2_(–CO_2_)_4_] in for example HKUST-1 with the underlying topology **tbo**.^36^ There are two problems, however.

The general deconstruction approach where a polynuclear coordination entity is contracted to one point obviously will contract any rod to a single point and thus reduce a 3D net to a 2D net, in this case the 6-connected **hxl**-net. The other issue is that the divisions we have done on the infinite polynuclear entity is arbitrary and difficult to generalize. In the approach giving the **soe**-net it is necessary to think of the net as constructed by polygons, triangles in this case, intersected by the rod. While the triangles are straight forward for this structure, this is not always the situation. This is not necessarily a problem from the analysis point of view, but the relation between theses single links, or the triangles as they are in our case, is lost. And the relation between the links projecting from the rod is completely determined by the topology of the rod. Thus, important structural information, like the existence of isomers, is potential lost if this approach is used. In this respect the problem is similar to the discussion of the “all node” deconstruction approach considering all branch points of the linkers, and the “single node” method considering only components mixed where the latter also will miss potential isomers.^14^

**Fig. 5:**
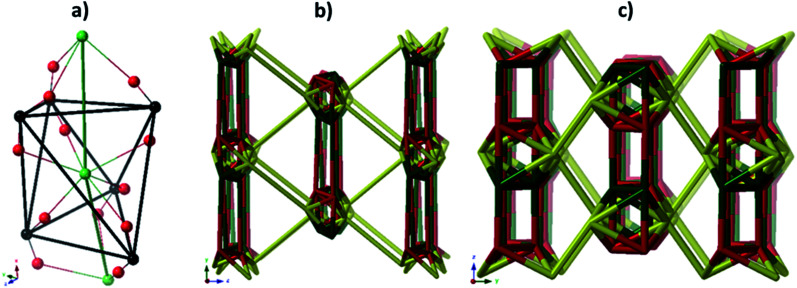
(a) The face sharing octahedra in the rod of JMS-1 (emphasized by green links) constructed from the carbonyl carbons (black tick lines). (b) The resulting **zaz**-net in the structure. (c) The most symmetric form of the **zaz**-net. This net has transitivity 3 12 (3 unique nodes and 12 unique edges).

As suggested by O'Keeffe and coworkers,^37^ we instead take the carbonyl carbons as the points of extension and create with them the simplest geometrical forms that describe the features of the rod. Often these descriptions turn out to be ladders, helices or linked polyhedra. In our case, the rod can be described as face-sharing octahedra, see Fig. 5.

**Fig. 6:**
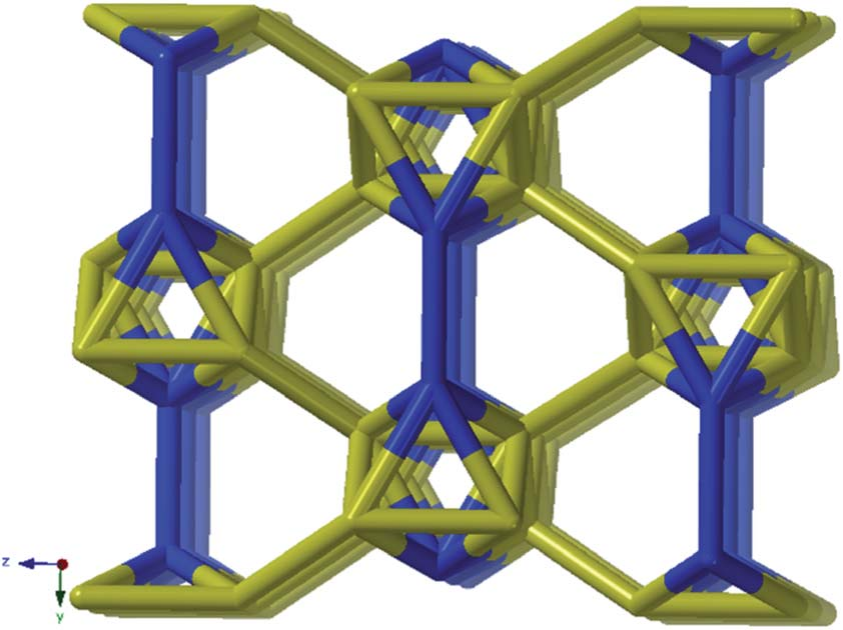
The **sct**-net based on face sharing octahedra, note that the number of links going out from the rod in all directions are the same. This net has transitivity 2 8 (2 unique nodes and 8 unique edges).

As every vertex in an octahedron is four-connected, face sharing the octahedra will mean every vertex is now six-connected, and bridging the rods with the ligands means we will have a seven-connected net. The most regular way of doing this is the **sct**-net, a pleasingly symmetric net where each octahedron have six links protruding from it with 60° angles if projected along the *a*-axis (Fig. 6).^32^ On the contrary, the **zaz**-net in the present structure have two pairs of two parallel links connecting the rods, resulting in a three-nodal net that is nevertheless symmetric and esthetically attractive.

### Functionalisation of JMS-1a

The catalyst Ru(ii)@JMS-1a was prepared as described in the experimental section. Comparison of the PXRD pattern of JMS-1a and JMS-1 suggest formation of a new phase upon activation. However, when JMS-1a is soaked in DMF for 24 h it reverts back to JMS-1 indicating a reversible structural transformation (Fig. 3). Upon cyclometalation, a new phase is formed as evidenced by the appearance of new diffraction peaks between which are not present in the parent JMS-1a indicative of framework flexibility. The FTIR (Fig. S2†) of JMS-1a and Ru(ii)@JMS-1a which have similar characteristic bands in the carboxylate stretching region. This observation confirms that the binding mode of the carboxylate moiety is not affected during cyclometalation of the JMS-1a.

### XPS analysis

**Fig. 7:**
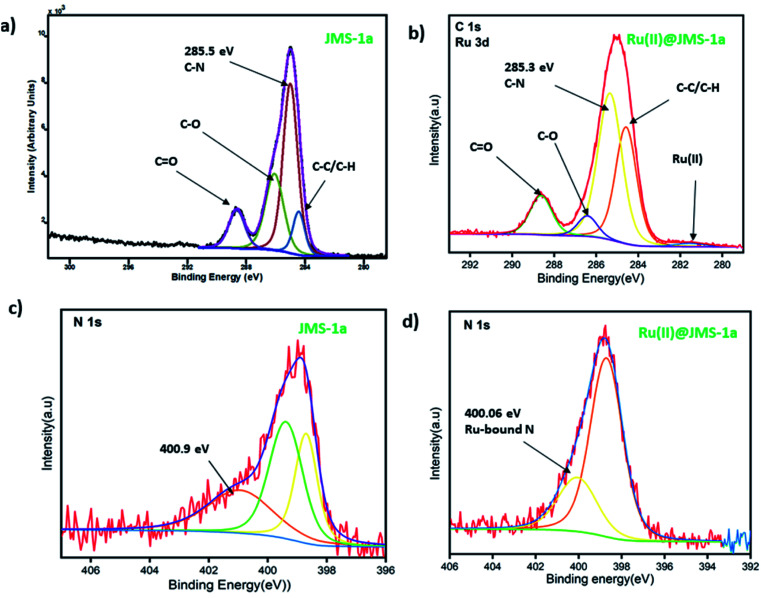
XPS spectra (a and b) C 1s (c and d) N 1s.

JMS-1a and Ru@JMS-1a were ground in order to evaluate the chemical state of the elements using XPS analysis. Initial state effects or chemical shifts, reflect the changes in the electronic structure of the material. Cyclometalation is a direct method to produce stable cyclometalated ruthenium(ii) complexes with [RuCl_2_(*p*-cymene)]_2_ precursor, *via* the C–H bond deprotonation in the presence of sodium acetate at room temperature in methanol.^38^ The cyclometalation of JMS-1a can easily be followed using XPS studies. During this reaction we expected the nitrogen and carbon of the linker to bind to the ruthenium(ii). Successful coordination of Ru(ii) to nitrogen and carbon should be reflected by a decrease in the binding energy carbon and nitrogen. Evidence of successful grafting of the Ru(ii) complex to the MOF is provided by the C 1s binding energy which shifts from 285.5 eV in JMS-1a to 285.3 eV in Ru(ii)@JMS-1a (Fig. 7a and b). The species indicated in Fig. 7 a and b is consistent with what is in the structure of JMS-1a and Ru(ii)@JMS-1a. In the case of N 1s binding energy (Fig. 7c and d), one identified peak is correlated with the change in the chemistry of JMS-1a. JMS-1a shows that the binding energy of N 1s is located at 400.9 eV. Upon cyclometalation with the Ru(ii) complex this energy shifts to 400.06 eV suggesting that the nitrogen atom donated electrons to the Ru(ii) ion. The observed shifts are consistent with low ruthenium loading content of 0.4% in Ru@JMS-1a.

### Sorption studies

**Fig. 8:**
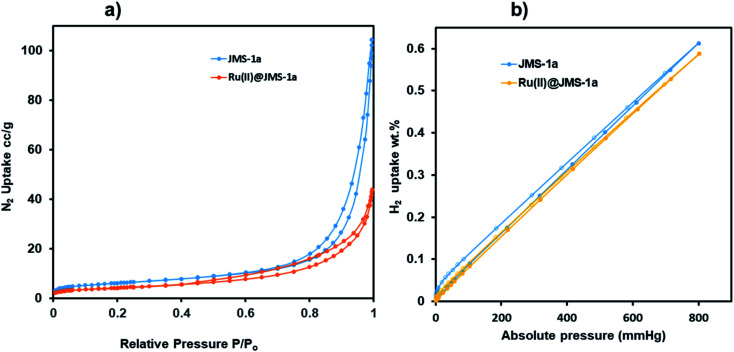
(a) Nitrogen and (b) hydrogen adsorption and desorption studies of JMS-1a and Ru(ii)@JMS-1a at 77 K.

Surface characterisation studies by BET (Fig. 8a) revealed a significant reduction in the BET surface are from 112 g m^−2^ to 58 g m^−2^ upon cyclometalation. The observed decrease in the surface area is ascribed to the blockage of the pores by the Ru(ii)*p*-cymene complex.

**Fig. 9:**
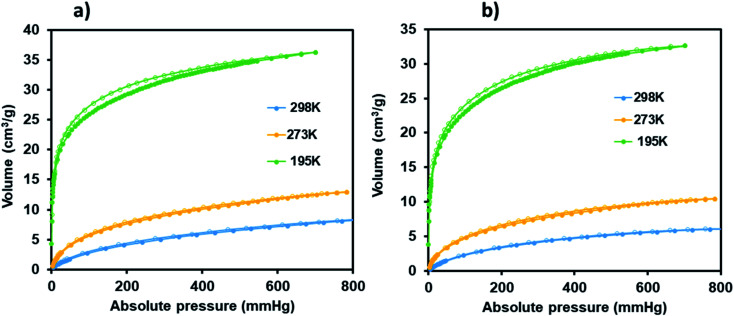
CO_2_ adsorption–desorption isotherms carried for JMS-1a (a) and Ru(ii)@JMS-1a (b) (closed circles represent adsorption and open circles desorption).

This is evidence of successful incorporation of Ru(ii)*p*-cymene within the pores of JMS-1a. The adsorption isotherms of JMS-1a and Ru(ii)@JMS-1a show type 1 isotherm at very low pressure (<0.4 *P*/*P*_o_) as illustrated in Fig. S3.† Similar behaviour was reported by Liang and co-workers.^39^ Hydrogen adsorption isotherms of JMS-1a and Ru(ii)@JMS-1a are depicted in Fig. 9b. At 77 K and 800 mmHg the aforementioned compounds can store up to 68.17 and 65.34 cm^3^ (STP) g^−1^, corresponding to sorption of 0.61 and 0.59 wt% respectively. Although the displayed hysteresis is rare,^40^ the H_2_ uptake is still comparable to several other MOFs reported in literature.^41–43^

Carbon dioxide sorption studies of JMS-1a and Ru(ii)@JMS-1a show a typical type 1 isotherms which suggests the microporosity nature of these materials (Fig. 9). JMS-1a has a volumetric uptake of 8.59 and 12.92 cm^3^ (STP) g^−1^ (0.384 and 0.576 mmol g^−1^) at 298 and 273 K respectively. The presence of Ru(ii)*p*-cymene in Ru(ii)@JMS-1a explains its reduced CO_2_ uptake of 6.08 and 10.42 cm^3^ (STP) g^−1^ (0.274 and 0.465 mmol g^−1^) at 298 and 273 K respectively. However, we noted that the amount of CO_2_ uptake by these material is comparable which is consistent with the structural modifications suggested by PXRD studies (Fig. 3). Cohen and co-workers found out that upon cyclometalation of the Zr based MOF with Ir complex, the amount of N_2_ uptake increased with increase in the Ir content relative to the unfunctionalised MOF.^44^ This was attributed to the framework flexibility although other mechanisms were not ruled out. At 195 K, the amount of CO_2_ uptake increases abruptly at low pressure range, with volumetric uptake more than doubling to 36.25 and 32.62 cm^3^ (STP) g^−1^ (1.61 and 1.46 mmol g^−1^) at an absolute pressure of 701 mmHg for JMS-1a and Ru(ii)@JMS-1a respectively. This confirms inherent permanent porosity of the MOFs. Carbon dioxide isotherms at 273 and 298 K reveal isosteric heats of adsorption (*Q*_st_) in the range 32 to 38 kJ mol^−1^ at load values ranging from 0.1 to 0.38 mmol g^−1^ indicating moderate to strong interaction of CO_2_ with the MOFs. The observed CO_2_ uptake are comparable to other MOFs reported in literature.^40,45–47^

### Catalysis

NMR analysis of the digested MOFs before was carried out to confirm that no DMF molecules in the MOF catalysts and the formate product observed in our reaction was not from the DMF molecules. Fig. S4† (a) to (c) shows the proton NMR of JMS-1a, [Ru(ii)Cl_2_(*p*-cymene)]_2_ in chloroform and the functionalised MOF Ru(ii)@JMS-1a. Coordination of the [Ru(ii)Cl(*p*-cymene)] to the activated phase JMS-1a is evidenced by 2 doublets around 5.3 ppm and 5.5 ppm (b) which shifted downfield and split into 4 doublets (c). The methyl protons of the *p*-cymene around 2.8 ppm, 2.1 ppm and 1.2 ppm shifted upfield upon coordination of Ru to the MOF backbone. Further proof is given by the splitting of the aromatic protons of the MOF, region 7.5 to 9.5 ppm (c). The absence of a peak at around 8 ppm confirms the absence of DMF in both JMS-1a and Ru(ii)@JMS-1a.

The conversion of CO_2_ to formate using JMS-1a and Ru(ii)@JMS-1a as catalysts was carried out as described in the experimental section. Table 2 shows the results that were obtained when JMS-1a and Ru(ii)@JMS-1a were used as catalysts for the conversion of carbon dioxide to formate under heterogeneous conditions. The actual mass of the MOF used for catalysis is presented in Table S1.† The formate formed was detected and quantified using ^1^H NMR (example 1 and Fig. S5 in ESI† shows how the yield of the formate was calculated).

**Table 2:** Catalytic performance of JMS-1a and Ru(ii)@JMS-1a^a^

Entry	Catalyst	Temp/°C	Ratio CO_2_/H_2_	Catalyst load (μmol)	Base	Solvent	Formate (mmol)	Yield/%
1	Ru(ii)@JMS-1a	90	1 : 3	15.6	KOH	THF	—	0
2	Ru(ii)@JMS-1a	90	1 : 3	15.6	KOH	Toluene	—	0
3	Ru(ii)@JMS-1a	90	1 : 3	15.6	KOH	Ethanol	2.07	41.4
4	Ru(ii)@JMS-1a	90	1 : 3	15.6	K_2_CO_3_	Ethanol	1.43	26.7
5	Ru(ii)@JMS-1a	90	1 : 3	15.6	NaHCO_3_	Ethanol	1.75	35.0
6	Ru(ii)@JMS-1a	90	1 : 3	15.6	Et_3_N	—	0.27	≈5
7	No catalyst	90	1 : 3	15.6	KOH	Ethanol	—	0
8	Ru(ii)@JMS-1a	90	1 : 3	15.6	No base	Ethanol	—	0
9	Ru(ii)@JMS-1a	110	0 : 4	15.6	KOH	Ethanol	—	0
10	Ru(ii)@JMS-1a	110	1 : 0	15.6	KOH	Ethanol	—	0
11	Ru(ii)@JMS-1a	110	1 : 4	15.6	KOH	Ethanol	4.77	95
12	Ru(ii)@JMS-1a	110	1 : 4	20.8	KOH	Ethanol	4.94	98.8
13	JMS-1a	110	1 : 4	15.6	KOH	Ethanol	3.10	62.0
14	[RuCl_2_(*p*-cymene)]_2_	110	1 : 4	15.6	KOH	Ethanol	1.22	24

a Time 24 h, amount of base 5 mmol. Yield based on ^1^H NMR analysis using acetone as an internal standard. The yield was calculated based on the conversion of 5 mmol added base.

Entry 1 and 2 shows that the solvent plays an important role in the formation of the product. The effect of the base was evaluated (entry 1 to 6). The highest yield of 41.4% was obtained using KOH base (entry 3) compared to 26.7 and 35.0% for K_2_CO_3_ and NaHCO_3_ respectively. It has been reported that high basicity is required for the deprotonation of the proton from metal dihydride complex (intermediate), and this explains why a high yield was obtained in the presence of KOH.^48^ Notably, the neutral amine base triethylamine (entry 6), which has been frequently employed in CO_2_ hydrogenation gave less satisfying results. This is because the Et_3_N molecule is bulky and cannot access the metal-hydride site to deprotonate the proton and this restricts catalysis on the surface of the MOF. This is evidence that the [Ru(ii)Cl(*p*-cymene)] complex in encapsulated inside the pores of the MOF. It was noted that in the absence of the solid catalyst (entry 7) and base (entry 8) no product was formed which confirms the importance of the catalyst and base in the reduction of CO_2_. Control experiments in the absence of CO_2_ and H_2_ was carried out to determine the source of carbon in the formate that was produced. Entry 9 and 10 shows that in the absence of the gases no product was formed confirming that DMF was not the source of formate. The activated phase JMS-1a's ability to convert CO_2_ was also evaluated, interestingly, ^1^H NMR studies revealed the formation of formate. However, we noted that under the same conditions the yield obtained using JMS-1a (62%, entry 13) was significantly lower than that of the functionalised material Ru(ii)@JMS-1a (entry 11). Majority of reports presented in literature use Zr-based MOFs that are functionalised with either Pt^16^ or Ir^15^ and none of these report on the activity of the Zr centre towards conversion of CO_2_ to formate. However, we are aware of the catalytic activities of lanthanides metals reported in literature.^45,49^ Having this in mind, we soaked our reactors in acid for 24 h and repeated the experiments several times using JMS-1a as the solid catalyst. These repeated experiments gave formate as the catalytic product, this confirmed that the La(iii) MOF act as a catalyst for reduction of CO_2_. As presented in Table 2, encapsulation of [Ru(ii)Cl(*p*-cymene)] gave rise to a significant increase in the product yield of formate. The combination of two metals in a single catalyst does not only lower the energetic requirements to speed up the reaction between carbon dioxide and hydrogen, but may possibly be altering the reaction pathway, to produce more desired product.

**Fig. 10:**
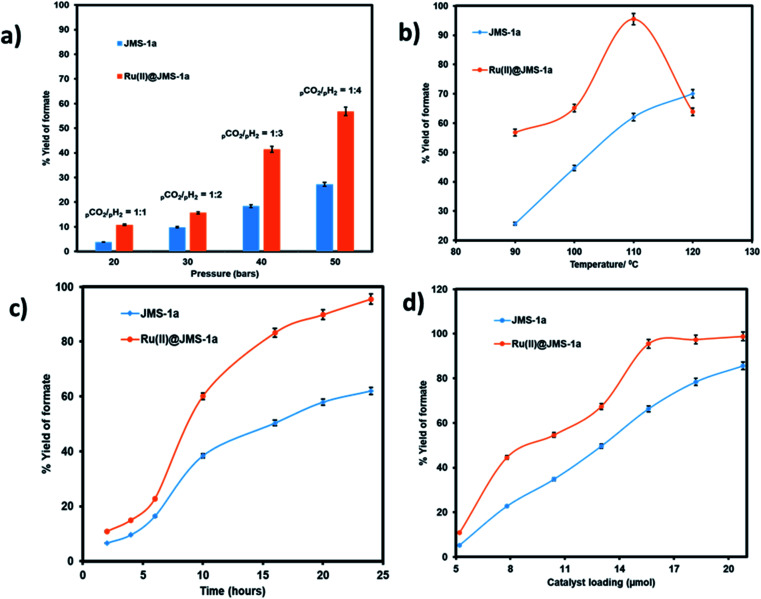
Effect of changing (a) the partial pressure of H_2_, (conditions: temperature 90 °C, time 24 hours, 15.6 μmol catalyst loading, 5 mmol and 8 mL ethanol), (b) temperature, (c) time, and (d) catalyst load: conditions for (b) to (d); (pressure 50 bar (CO_2_/H_2_ = 1 : 4), temperature 110 °C, time 24 hours, 5 mmol KOH and 8 mL ethanol on the yield of formate produced.

We evaluated the effect pressure, temperature, time and catalyst loading on the formation of the formate using the two catalysts (Fig. 10). Pressure variation was determined by changing the partial pressure of H_2_ while keeping the pressure of CO_2_ constant.

As illustrated in Fig. 10a an increase in the partial pressure of H_2_ results in an increase in the yield of formate produced by both JMS-1a and Ru(ii)@JMS-1a catalysts. On increasing the pressure of H_2_ to 40 bars while keeping the CO_2_ pressure at 10 bars, the yield of formate produced increased up to 56% and 27.3% for Ru(ii)@JMS-1a and JMS-1a respectively. This is attributed to the fact that sufficient partial pressure of H_2_ is required to generate the hydride and boost CO_2_ hydrogenation. The effect of temperature on the formation of formate was carried out at, 90 °C, 100 °C, 110 °C and 120 °C with other parameters being kept constant (Fig. 10b). We observed that the conversion of CO_2_ increases gradually with an increase in temperature reaching a yield of 95% at 110 °C for Ru(ii)@JMS-1a. At 120 °C, a dramatic decrease in the yield of formate produced maybe be due to the decomposition of the formate species or the departure of the *p*-cymene molecule from the MOF backbone. Contrary to Ru(ii)@JMS-1a which shows a dramatic decrease in the yield of formate produced at 120 °C, JMS-1a shows a gradual increase in yield of formate as temperature is increased although the yield of formate produced is lower than that of the functionalised MOF. Fig. 10c shows how the yield of formate produced varies with time. The yield increases with an increase in time to reach a yield of 94% and 62% for Ru(ii)@JMS-1a and JMS-1a respectively. The effect of the catalyst load was also evaluated for both catalysts (Fig. 10d). In both cases, an increase in the catalyst load gave rise to an increase in the amount of formate produced which indicates that the formation of formate is 1st order with respect to the catalyst.^50^ When 18.2 μmol of Ru(ii)@JMS-1a catalyst was used, 4.87 mmol of formate was produced which corresponds to 97% yield. The activated MOF JMS-1a produced 4.57 mmol of formate (91% yield) when 20.8 μmol of catalysts was used. The ruthenium precursor used for functionalization of the MOF was also employed in the conversion of CO_2_ to formate under optimised conditions. A yield of 24.3% formate was produced which is significantly lower than that of JMS-1a and Ru(ii)@JMS-1a.

**Fig. 11:**
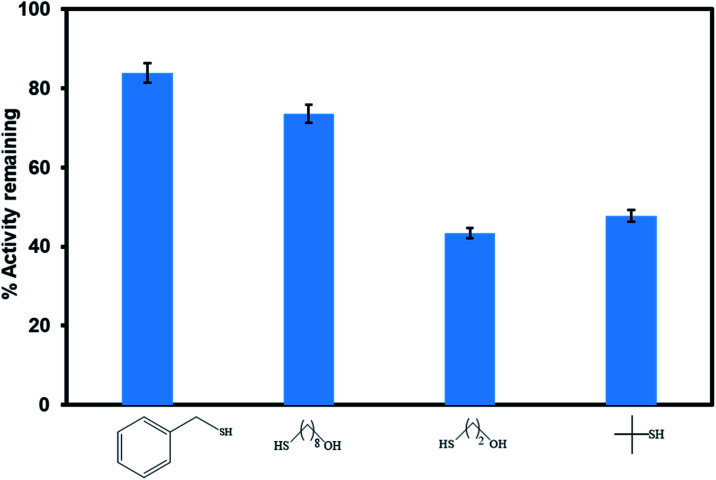
Comparison of the catalytic activity of Ru(ii)@JMS-1a in the presence of different sized thiol poisons.

Poisoning studies were carried out using a wide range of different sized thiols to prove successful incorporation of the [Ru(ii)Cl(*p*-cymene)] inside the pores of the MOF rather than on the surface (Fig. 11). Thiols are known to poison many transition metal catalysts. When bulky thiols benzylmercaptan and 8 mercapto-1-octanol were exposed to Ru(ii)@JMS-1a the product yield was reduced by 16% and 24% respectively. In contrast, when Ru(ii)@JMS-1a exposed to 2 mercapto-ethanol and 2 methyl-2-propanethiol, the yield was reduced by 56.6 and 52.2% respectively. Fig. S6–S9† shows the ^1^H NMR spectra of these studies. The absence of appreciable inhibition in the presence of the bulky thiols suggests that the most of the active species is encapsulated in the framework rather than bound to the surface. Notably, the most effective poisons are the least sterically demanding thiols (2 mercapto-ethanol and 2 methyl-2-propanethiol). This observation is consistent with bulk of the catalyst being encapsulated inside the MOF pores because the smaller thiols can easily access the catalytic sites by diffusion through the pores and poison the catalyst. Tsung and co-workers reported similar findings.^51^

### Leaching, heterogeneity, and recycling studies

To investigate whether Ru(ii)@JMS-1a and JMS-1a were actually working in a heterogeneous manner, the solid catalyst was filtered over a reaction duration of 6 h (for which a formate (1.14 mmol for Ru(ii)@JMS-1a and 0.82 mmol for JMS-1a) was observed in the filtrate); the resulting colourless filtrate was used as the catalytic solution. Even after an extended period of time, no increase in formate was observed, whereas the original reaction produced a 4.8 and 3.10 mmol of formate for Ru(ii)@JMS-1a and JMS-1a respectively after 24 h. This result suggests that the catalyst does not leach into the solution, and that the catalysts functions as a supported molecular catalyst. ICP-OES analysis of the aqueous solution in the reactor showed negligible metal leaching (0.0001% of La and <0.0001% of Ru for Ru(ii)@JMS-1a and 0.0001% La for JMS-1a).

**Fig. 12:**
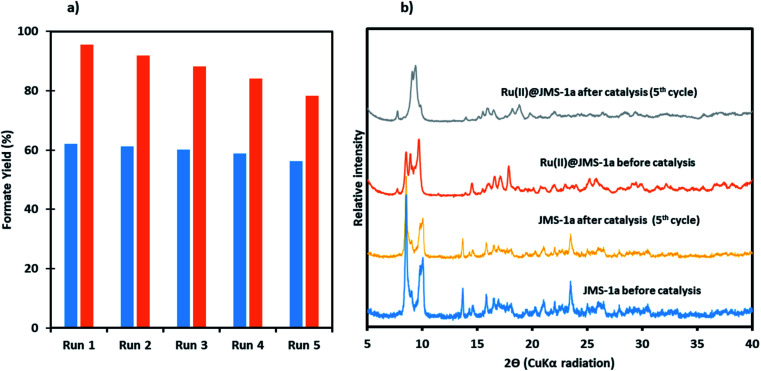
A comparison of the performance of catalyst JMS-1a and Ru@JMS-1a over 5 consecutive cycles, (a) yield of formate produced (blue JMS-1a and orange Ru(ii)@JMS-1a), (b) PXRD studies of the catalysts before and after catalysis.

To demonstrate recyclability of the prepared catalysts, their use over multiple runs was studied. For this, the hydrogenation was initially performed in a 5 mmol KOH solution at 110 °C under a total pressure of 50 bars for 24 h with a catalyst load of 20.8 μmol and 15.6 μmol for Ru(ii)@JMS-1a and JMS-1a respectively. After the initial run, the catalysts were separated by filtration, washed thoroughly with ethanol and dried. The dried catalysts were then directly used for the next run with a fresh 5 mmol KOH solution. As shown in Fig. 12a, with JMS-1a, the yield of formate produced remained almost constant over the 5 runs. Contrary to this observation, Ru(ii)@JMS-1a showed 17% decrease in the yield over five consecutive cycles although the yield obtained (78%) was still higher than that obtained using JMS-1a. PXRD studies Fig. 12b of the recycled catalyst after 5 cycles show that the structural integrity of JMS-1a is retained while that of Ru(ii)@JMS-1a is slightly changed. Furthermore, the FTIR studies (Fig. S10†) before and after catalysis of the MOFs shows that the symmetric and asymmetric carboxylate stretches are located at similar positions. TGA analysis presented in Fig. S11† shows similar thermal profiles of the MOFs before and after catalysis.

### Mechanisms

**Scheme 1:**
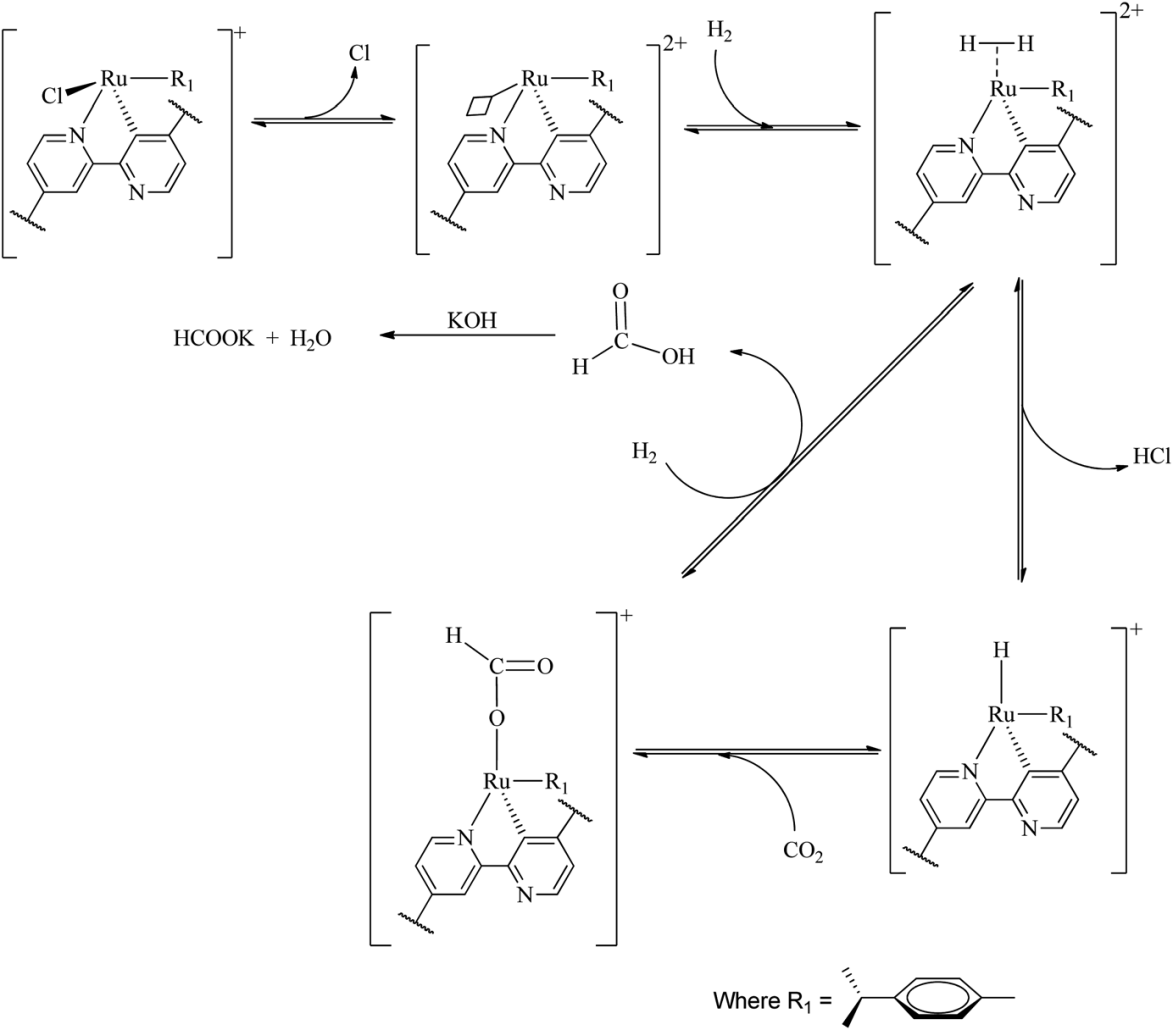
Plausible reaction mechanism for the hydrogenation of CO_2_ with Ru(ii)@JMS-1a.

A large number of different reaction mechanisms have then been proposed for the metal-hydride reaction with either the hydrolyzed (HCO_3_^−^) or original CO_2_. They all have in common nucleophilic attack of the hydride ligand on the carbon atom in the substrate. This study does not contribute any new understanding of the mechanism(s) for the reduction of CO_2_ to formate at the La(iii) sites and we refer the reader to the relevant literature.^52^ Both outer sphere,^53^ and inner sphere^54^ reactions of CO_2_/HCO_3_^−^ have been proposed. The formation of formate at the Ru(ii) sites is proposed as follows (Scheme 1), the first step involves reduction elimination of the Cl. This step provides open metal sites for the coordination of H_2_ molecules to give a dihydrogen complex [Ru(H_2_) (*p*-cymene)]. The active species, the ruthenium hydride RuH(*p*-cymene) is formed upon splitting of the coordinated H_2_ molecules with simultaneous elimination HCl. The insertion of CO_2_*via* associative addition into the ruthenium hydride complex generates the formate complex which readily dissociates the formate.

## Conclusion

MOF JMS-1 has been successfully prepared and structurally characterised using spectroscopic and diffraction techniques. PXRD studies revealed a phase change induced by inclusion of the [Ru(ii)Cl(*p*-cymene)] in JMS-1a. Hydrogenation of CO_2_ studies using the activated material (JMS-1a) and the functionalised MOF (Ru(ii)@JMS-1a) revealed that both catalysts were able to convert CO_2_ to formate. However, the Ru(ii) functionalised MOF produced high yields of formate in comparison to JMS-1a. Ru@JMS-1a performed better at temperatures below 110 °C while JMS-1a required harsh conditions to produce high yield of formate. Remarkably, JMS-1a can be easily recycled for five cycles without significant loss in the yield of formate while Ru(ii)@JMS-1a shows a slight decrease in the yield of the product. This work is important as it present a new strategy of incorporating catalytically active centres in the MOF through cyclometalation. We have also demonstrated for the first time that La(iii) MOF can be used for conversion of CO_2_ to formate. In future we expect to modulate the properties of the linker so that we can obtain a better yield of the product under less harsh conditions using La(iii) MOFs.

## Conflicts of interest

There are no conflicts of interest to declare.

## Supplementary Material

RA-010-C9RA09938G-s001

RA-010-C9RA09938G-s002
