# Supplementary material for: Cyclometalation of lanthanum(iii) based MOF for catalytic hydrogenation of carbon dioxide to formate
Source: RSC Adv. 2020 Jan 22;10(6):3593–605. doi: 10.1039/c9ra09938g (PMC9048731; doi:10.1039/c9ra09938g)
Supplement: RA-010-C9RA09938G-s001 [file RA-010-C9RA09938G-s001.pdf]

Electronic Supplementary Material (ESI) for RSC Advances.
This journal is © The Royal Society of Chemistry 2020

Cyclometalation of Lanthanum(III) based MOF for Catalytic Hydrogenation of Carbon Dioxide to Formate:

Piwai Tshuma,[ab] Banothile Makhubela[b] Lars Ohmstrom[c] Susan A Bourne[d] and Gift Mehlana[a]*

[a]Midlands State University, Department of Chemical Technology, Faculty of Science and Technology, Private Bag 9055, Senga Road, Gweru, Zimbabwe

[b]University of Johannesburg, Department of Chemistry, Faculty of Science, Kingsway Campus: C2 Lab 328, Auckland Park, 2006, South Africa

[c]Chalmers University of Technology, Department of Chemistry and Chemical Engineering

Physical Chemistry room 9029, Göteborg, Sweden

[d]University of Cape Town, Department of Chemistry, Faculty of Science, PD Hahn Building 7701 Rondebosch, Cape Town, South Africa

E-mail Address: mehlanag@staff.msu.ac.zw

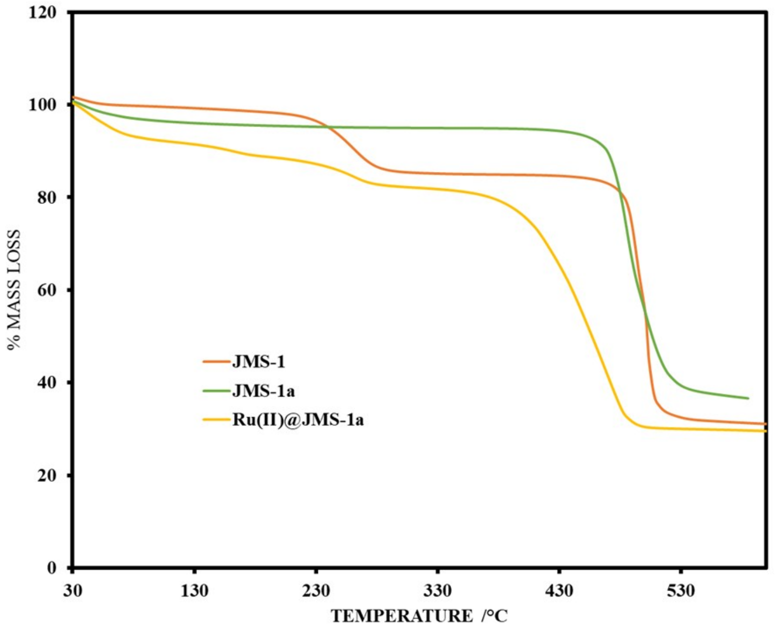

Figure S1: TGA traces of the as made JMS-1, activated JMS-1a and Ru(II)@JMS-1a

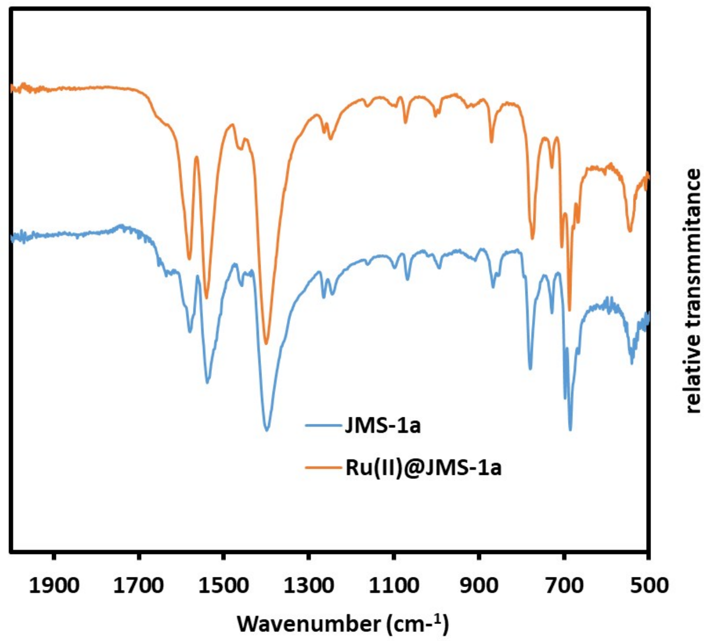

Figure S2: FTIR studies of JMS-1a and Ru(II)@JMS-1a showing characteristic carboxylate stretches

located at the same positions.

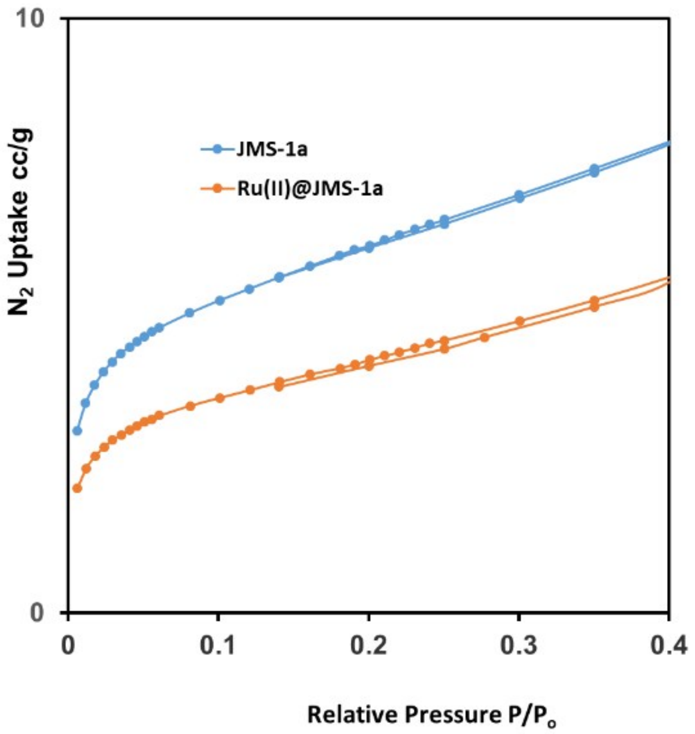

Figure S3: Nitrogen adsorption and desorption studies at 77K for JMS-1a and Ru(II)@JMS-1a

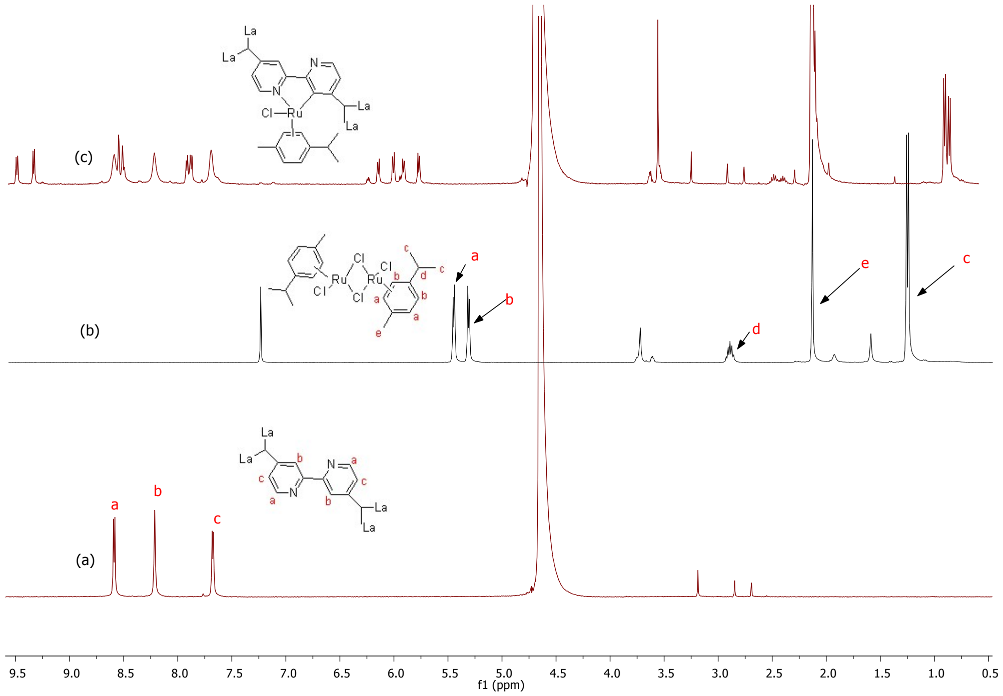

Figure S4. ^{1}HNMR of (a) JMS-1a in D_2O, (b) $[RuCl_2\text{-}p\text{cymene}]_2$ and (c) Ru(II)@JMS-1a in D_2O.

Example 1, Entry 3 (Table 2)

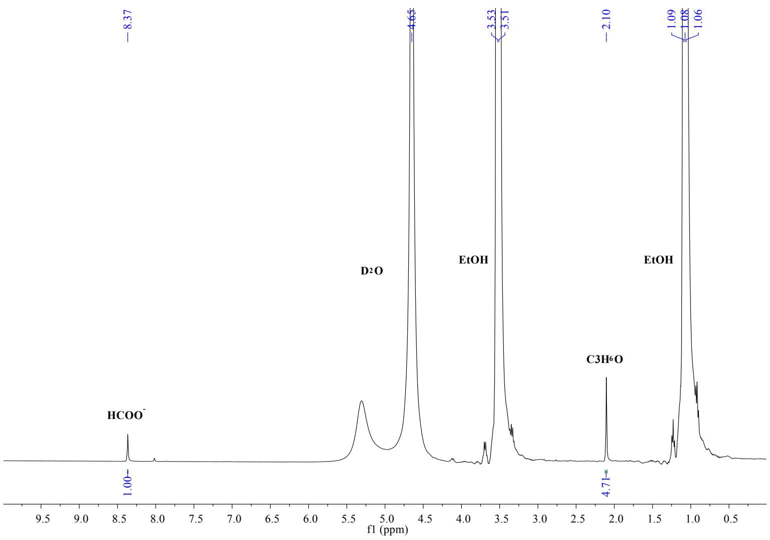

Figure S5: NMR of entry 3 (Table 2)

Amount of sample after catalysis for NMR = 0.2 mL

NMR solvent, D_2O = 0.3 mL

Volume of ethanol used for catalysis = 8.0 mL

Moles of Standard = 0.0000405836 calculated from density and volume of standard used (3μL)

The product potassium formate has 1 proton, so we divide the integral value for fromate by 1. Acetone, the internal standard has 6 protons, so divide the integral value for acetone by 6.

Acetone = 4.71/6 = 0.785

Formate = 1/1 = 1

Number of moles of formate = (0.0000405836)/(0.785/1)

= 0.0000517 mols in 0.2mL product

Moles in 8 mL = 0.00207 mol

Yield = (moles of formate produced/moles of KOH) * 100%

= (0.00207/0.005)

= 41.4%

Table S1: Catalytic performance of JMS-1a and Ru(II)@JMS-1a showing the actual mass of MOF used in mg

Entry	Catalyst	Temp/ °C	Ratio CO_2/H_2	Mass of MOF used/mg	Base	Solvent	Formate (mmol)	Yield/ %
1	Ru(II)@JMS-1a	90	1:3	31.5	KOH	THF	-	0
2	Ru(II)@JMS-1a	90	1:3	31.5	KOH	Toluene	-	0
3	Ru(II)@JMS-1a	90	1:3	31.5	KOH	Ethanol	2.07	41.4
4	Ru(II)@JMS-1a	90	1:3	31.5	K_2CO_3	Ethanol	1.43	26.7
5	Ru(II)@JMS-1a	90	1:3	31.5	$NaHCO_3$	Ethanol	1.75	35.0
6	Ru(II)@JMS-1a	90	1:3	31.5	Et_3N	-	0.27	≈5
7	No catalyst	90	1:3	31.5	KOH	Ethanol	-	0
8	Ru(II)@JMS-1a	90	1:3	31.5	No base	Ethanol	-	0
9	Ru(II)@JMS-1a	110	0:4	31.5	KOH	Ethanol	-	0
10	Ru(II)@JMS-1a	110	1:0	31.5	KOH	Ethanol	-	0
11	Ru(II)@JMS-1a	110	1:4	31.5	KOH	Ethanol	4.77	95
12	Ru(II)@JMS-1a	110	1:4	41.8	KOH	Ethanol	4.94	98.8
13	JMS-1a	110	1:4	15.9	KOH	Ethanol	3.10	62.0

Poisoning studies

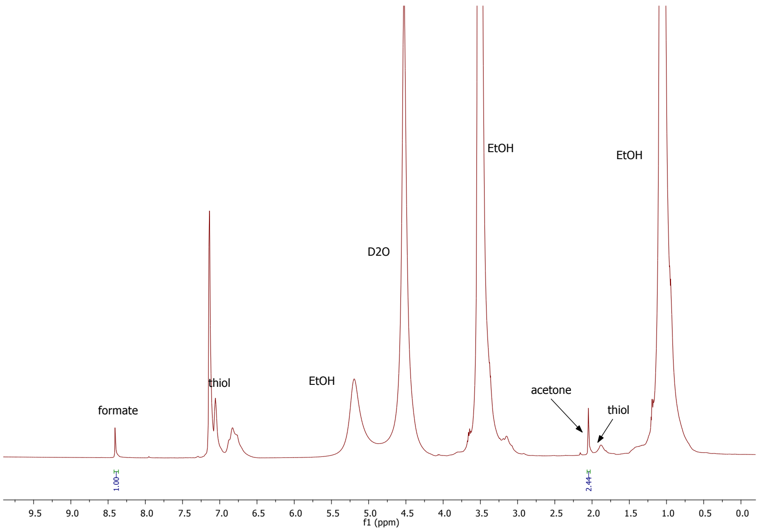

Figure S6. Catalysis in the presence of benzylmercaptan

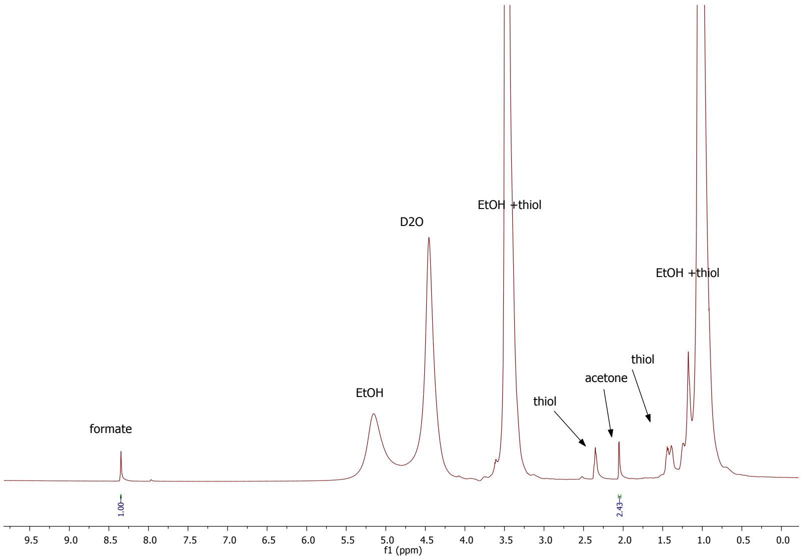

Figure S7. Catalysis in the presence of 8 mercapto-1-octanol

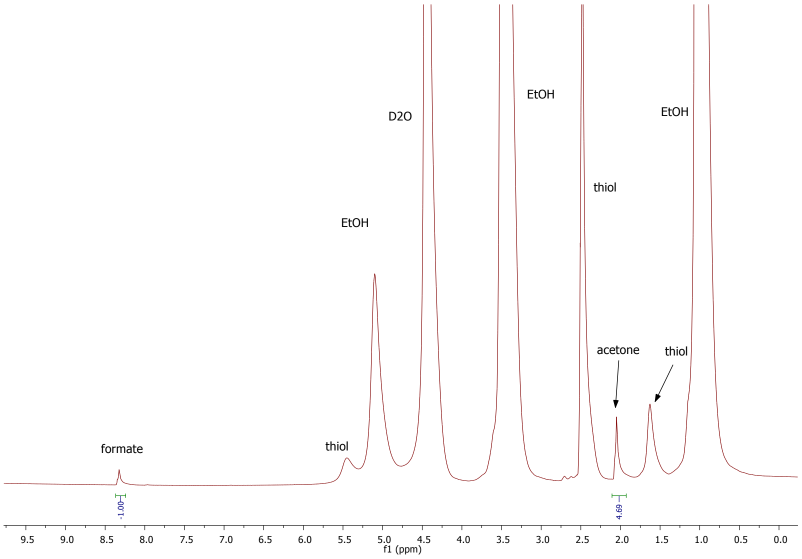

Figure S8 Catalysis in the presence of 2 mercapto-ethanol

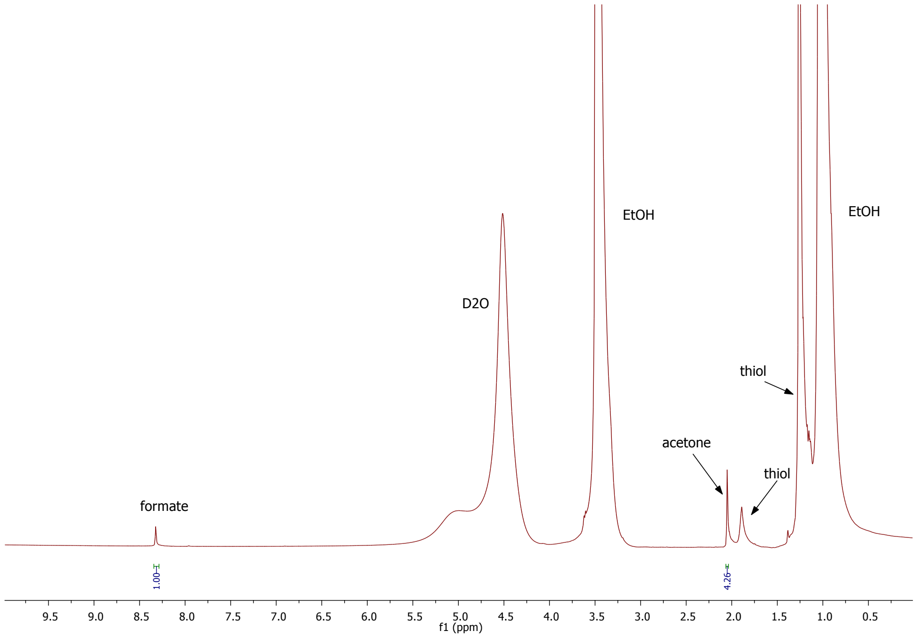

Figure S9: Catalysis in the presence of 2 methyl 2 propanethiol

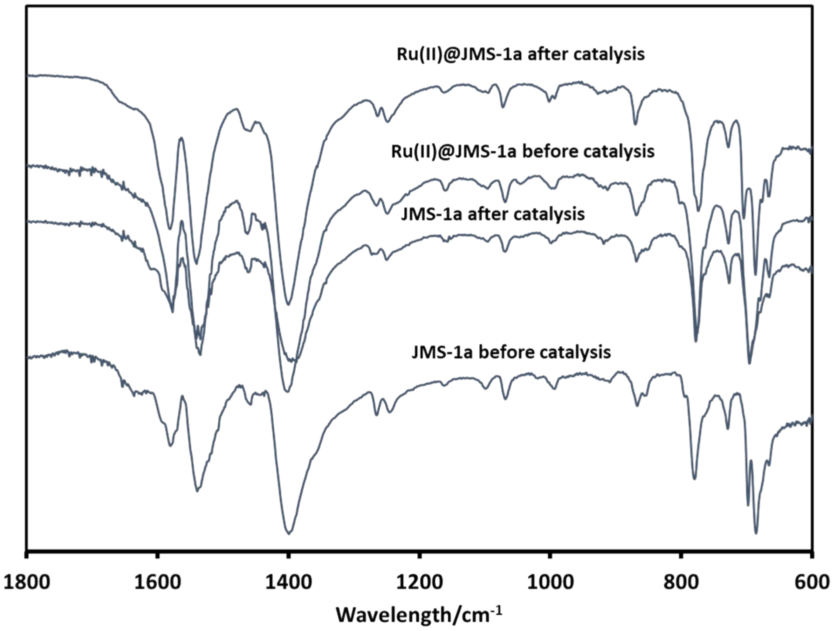

Figure S10: FTIR studies of JMS-1a and Ru(II)@JMS-1a showing characteristic carboxylate asymmetric and symmetric stretches located in similar positions.

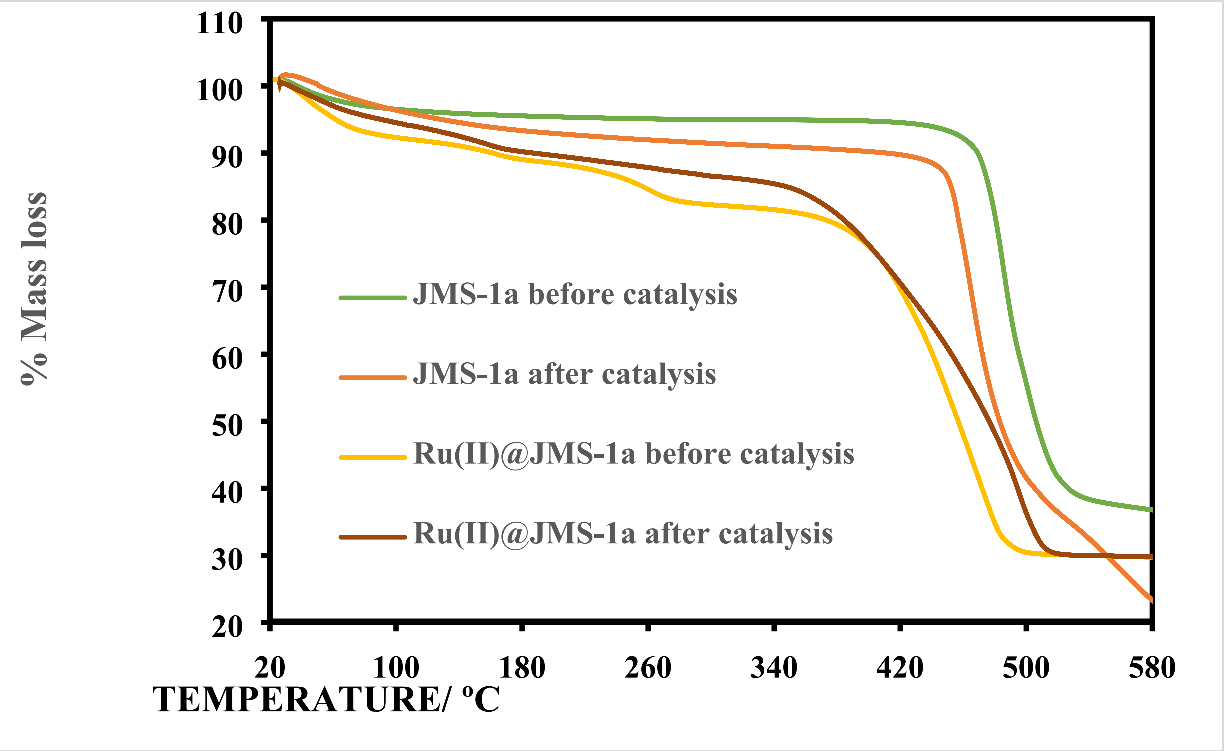

Figure S11: A comparison of the thermal profiles of the MOFs before and after catalysis
